# Comparison of First- and Second-Generation Drug-Eluting Stents in Patients with Acute Myocardial Infarction and Prediabetes Based on the Hemoglobin A1c Level

**DOI:** 10.1155/2020/1710439

**Published:** 2020-07-18

**Authors:** Yong Hoon Kim, Ae-Young Her, Myung Ho Jeong, Byeong-Keuk Kim, Sung-Jin Hong, Seunghwan Kim, Chul-Min Ahn, Jung-Sun Kim, Young-Guk Ko, Donghoon Choi, Myeong-Ki Hong, Yangsoo Jang

**Affiliations:** ^1^Division of Cardiology, Department of Internal Medicine, Kangwon National University School of Medicine, Chuncheon, Republic of Korea; ^2^Department of Cardiology, Chonnam National University Hospital, Gwangju, Republic of Korea; ^3^Division of Cardiology, Severance Cardiovascular Hospital, Yonsei University College of Medicine, Seuol, Republic of Korea; ^4^Division of Cardiology, Inje University College of Medicine, Haeundae Paik Hospital, Busan, Republic of Korea

## Abstract

**Objective:**

To compare major clinical outcomes after successful percutaneous coronary intervention (PCI) with first-generation (1G) drug-eluting stents (DES) and second-generation (2G) DES in patients with acute myocardial infarction (AMI) and prediabetes.

**Background:**

Patients with prediabetes are associated with an increased incidence of coronary artery disease. The relative superiority of 1G- and 2G-DES in these patients is not well established.

**Methods:**

A total of 4997 patients with AMI and prediabetes were divided into two groups: the 1D-DES group (*n* = 726) and the 2G-DES group (*n* = 4271). The primary outcomes were the patient-oriented composite outcomes (POCOs) defined as all-cause death, recurrent myocardial infarction (Re-MI), and any disease revascularization at 2-year follow-up. The secondary outcome was probable or definite stent thrombosis (ST).

**Results:**

After propensity score-matching (PSM) analysis, two PSM groups (698 pairs, *n* = 1396, C-statistics = 0.725) were generated. The cumulative incidence rates of POCOs (hazard ratio (HR): 1.467; 95% confidence interval (CI): 1.068–2.015; *p* = 0.018), any disease revascularization (HR: 2.259; 95% CI: 1.397–3.654; *p* = 0.001), and ST (HR: 4.361; 95% CI: 1.243–15.30; *p* = 0.021) in the 1G-DES group were significantly higher than those in the 2G-DES group. However, the cumulative incidence rates of all-cause death, cardiac death, and Re-MI were similar between the two groups.

**Conclusions:**

In patients with AMI and prediabetes, 2G-DES implantation was more efficacious than 1G-DES implantation over a 2-year follow-up period. However, further studies are needed to confirm these results.

## 1. Introduction

In the fibrinolytic era, hyperglycemia, rather than normoglycemia, was a major independent prognostic factor of adverse clinical outcomes in patients with acute myocardial infarction (AMI) [1, 2]. Similarly, in the drug-eluting stent (DES) era, hyperglycemia is an independent predictor of early and late mortality in patients with ST-segment elevation myocardial infarction (STEMI) regardless of the presence or absence of known diabetes [3, 4]. The exact mechanisms by which hyperglycemia is associated with adverse major clinical outcomes in AMI have not been fully elucidated yet. With regard to diabetes mellitus (DM), many studies have shown its association with higher long-term risk of death, myocardial infarction (MI), and repeat revascularization in patients undergoing percutaneous coronary intervention (PCI) [5–7]. Patients with prediabetes are at an increased risk of cardiovascular disease (CVD), and prediabetes is associated with an increased incidence of coronary artery disease (CAD) [8, 9]. Even though the relative superiority between the first-generation (1G) and second-generation (2G) DESs in patients with AMI is controversial [10–12], most previous studies were not performed under the circumstance of prediabetes. Therefore, the comparative long-term clinical outcomes between the two DES generations were limited. Hence, we investigated and compared the major clinical outcomes after successful PCI with 1G-DES and 2G-DES in patients with AMI and prediabetes over a 2-year follow-up period.

## 2. Methods

### 2.1. Study Population

**Figure 1:**
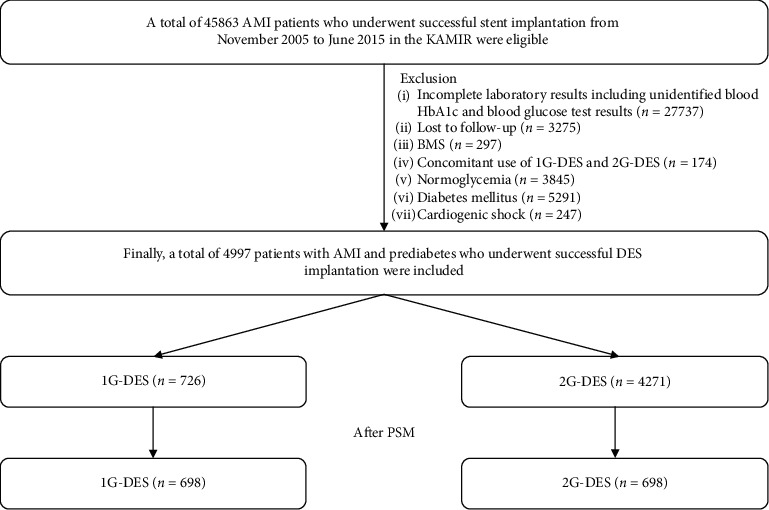
Study flowchart. AMI: acute myocardial infarction; KAMIR: Korea AMI Registry; HbA1c: hemoglobin A1c; BMS: bare-metal stent; 1G: first-generation; 2G: second-generation; DES: drug-eluting stent; PSM: propensity score-matching analysis.

This study was a nonrandomized, multicenter, observational, and retrospective cohort study. A total of 45863 patients from the Korea AMI Registry (KAMIR) who had AMI and underwent successful stent implantation between November 2005 and June 2015 were evaluated. KAMIR is the first nationwide and multicenter registry that included >50 community and teaching hospitals in South Korea since November 2005 [13]. Eligible patients were aged ≥18 years at the time of hospital admission. Among the patients, those with incomplete laboratory results, including unidentified blood hemoglobin (Hb) A1c and blood glucose test results (*n* = 27737, 60.5%), those who were lost to follow-up (*n* = 3275, 7.1%), those who received a bare-metal stent (*n* = 297, 0.6%), those with concomitant use of 1G-DES and 2G-DES (*n* = 174, 0.4%), those with normoglycemia (*n* = 3845, 8.4%), those with DM (*n* = 5291, 11.5%), and those with cardiogenic shock (*n* = 247, 0.5%) were excluded. Finally, 4997 patients with AMI and prediabetes who underwent successful DES implantation were included in the study. The patients were divided into two groups: the 1G-DES group (*n* = 726, 14.5%; sirolimus-eluting stent (SES; Cypher, Cordis Corp., Miami Lakes, Florida; *n* = 313, 43.1%) and paclitaxel-eluting stent (PES, Taxus, Boston Scientific, Natick, Massachusetts; *n* = 413, 56.9%)) and the 2G-DES group (*n* = 4271, 85.5%; zotarolimus-eluting stent (ZES; Resolute Integrity, Medtronic, Inc., Minneapolis, MN; *n* = 1466, 34.3%), everolimus-eluting stent (EES; Xience Prime, Abbott Vascular, Santa Clara, CA; or Promus Element, Boston Scientific, Natick, MA; *n* = 2132, 49.9%), biolimus-eluting stent (BES; BioMatrix Flex, Biosensors International, Morges, Switzerland; or Nobori, Terumo Corporation, Tokyo, Japan; *n* = 577, 13.5%), and other stents (*n* = 96, 2.2%); Figure 1). The study protocol was approved by the institutional review board of each participating center, and the study was conducted in accordance with the principles of the 1975 Declaration of Helsinki. In this retrospective study, we evaluated patients who had provided written informed consent prior to participation in the KAMIR study. Therefore, any information concerning adverse events of these 4997 participants with AMI and prediabetes including the time intervals and the types of events after the index PCI, which occurred during a 2-year follow-up period, was monitored at the outpatient clinic, by phone calls, or by reviewing their charts at each participating center in those days.

### 2.2. Percutaneous Coronary Intervention and Medical Treatment

Coronary angiography and PCI were performed in accordance with the general guideline [14]. Before PCI, all the patients received loading doses of aspirin 200–300 mg and clopidogrel 300–600 mg; alternatively, ticagrelor 180 mg or prasugrel 60 mg was administered. Dual antiplatelet therapy (DAPT; a combination of aspirin 100 mg/day with clopidogrel 75 mg/day or ticagrelor 90 mg twice daily or prasugrel 5–10 mg/day) for > 12 months was recommended for patients who underwent PCI. Administration of triple antiplatelet therapy (cilostazol (Pletal®, Otsuka Pharmaceutical Co., Tokyo, Japan) 100 mg twice daily added to DAPT) was left to the discretion of the individual physicians.

### 2.3. Study Definitions and Clinical Outcomes

Prediabetes was determined based on the medical history and glycated hemoglobin (HbA1c) and fasting plasma glucose (FPG) levels at the index hospitalization and defined as an HbA1c level of 5.7%–6.4% and an FPG level of 100–125 mg/dL (5.6–6.9 mmol/L) [15]. ST-segment elevation myocardial infarction (STEMI) and non-STEMI were defined in accordance with the current guidelines [16, 17]. In NSTEMI cases, an early invasive treatment strategy was defined as performing PCI within 24 hours after admission [17]. A successful PCI was defined as a residual stenosis of <30% and thrombolysis in myocardial infarction (TIMI) with grade III flow for the infarct-related artery (IRA) after the procedure. The primary clinical outcome of this study was the occurrence of patient-oriented composite outcomes (POCOs) defined as all-cause death, recurrent myocardial infarction (Re-MI), or any disease revascularization (ADR) at 2-year follow-up. The secondary outcome was definite or probable stent thrombosis (ST) at 2-year follow-up. All-cause death was classified as cardiac death (CD) or non-CD. ADR was composed of target lesion revascularization (TLR), target vessel revascularization (TVR), and non-TVR. The definitions of Re-MI, TLR, TVR, and non-TVR were previously published [18]. The cumulative incidence of ST was defined by current consensus as acute (0 to 24 h), subacute (24 h to 30 days), late (30 days to 1 year), and very late (>1 year) [19].

### 2.4. Statistical Analyses

For continuous variables, in this study, the normality test was performed using the Kolmogorov–Smirnov normality test. According to the normality results, the independent samples *t*-test was applied to examine the difference of continuous variables means of the two groups, and the data were expressed as the mean ± standard deviations. For categorical variables, the differences between the two groups were analyzed using the chi-squared test or, if not applicable, Fisher's exact test, and the data were expressed as counts and percentages. To adjust for potential confounders, a propensity score-matching (PSM) analysis was performed using a logistic regression model. We tested all available variables listed in Table 1 that could be of potential relevance. The C-statistics for PSM was 0.725 in the current study. Patients in the 1G-DES group were then one-to-one matched to those in the 2G-DES group according to propensity scores with the nearest available pair-matching method. Subjects were matched with a caliper width equal to 0.01. The procedure yielded 698 matched pairs. Various clinical outcomes were estimated using the Kaplan–Meier method, and differences between the two groups were compared using the log-rank test. For all analyses, two-sided values of *p* < 0.05 were considered statistically significant. All statistical analyses were performed using the SPSS version 20 software (IBM; Armonk, NY, USA).

## 3. Results

### 3.1. Baseline Characteristics

The baseline clinical, laboratory, and procedural characteristics of the study population are summarized in Table 1. The mean age (63.8 ± 12.1 years vs. 64.2 ± 12.4 years, *p*=0.464) and proportion of men (73.7% vs. 74.2%, *p*=0.773) of the enrolled patients were similar between the two groups. The mean left ventricular ejection fraction (LVEF) was not significantly different between the two groups (52.6% ± 12.4% vs. 52.4% ± 11.2%, *p*=0.798) and was >50% in both groups. However, the proportion of patients who underwent primary PCI (92.8% vs. 96.1%, *p*=0.003) or PCI within 24 hours (76.6% vs. 85.5%, *p* < 0.001) was significantly higher in the 2G-DES group than in the 1G-DES group. The proportion of patients with cardiopulmonary resuscitation (CPR) on admission (4.2% vs. 2.3%, *p*=0.013) and the prescription rates of aspirin, ticagrelor, prasugrel, and lipid-lowering agents as discharge medications; the number of American College of Cardiology/American Heart Association (ACC/AHA) type C lesions; the incidence of single-vessel disease; the use frequency of optical coherence tomography and fractional flow reserve; and the mean total length of the deployed stents were significantly greater in the 2G-DES group than in the 1G-DES group. By contrast, the prescription rates of clopidogrel and cilostazol as discharge medications and the incidence of ≥ 3-vessel disease were significantly higher in the 1G-DES group than in the 2G-DES group. However, these intergroup differences in baseline characteristics were well balanced after PSM adjustment.

### 3.2. Clinical Outcomes

**Figure 2:**
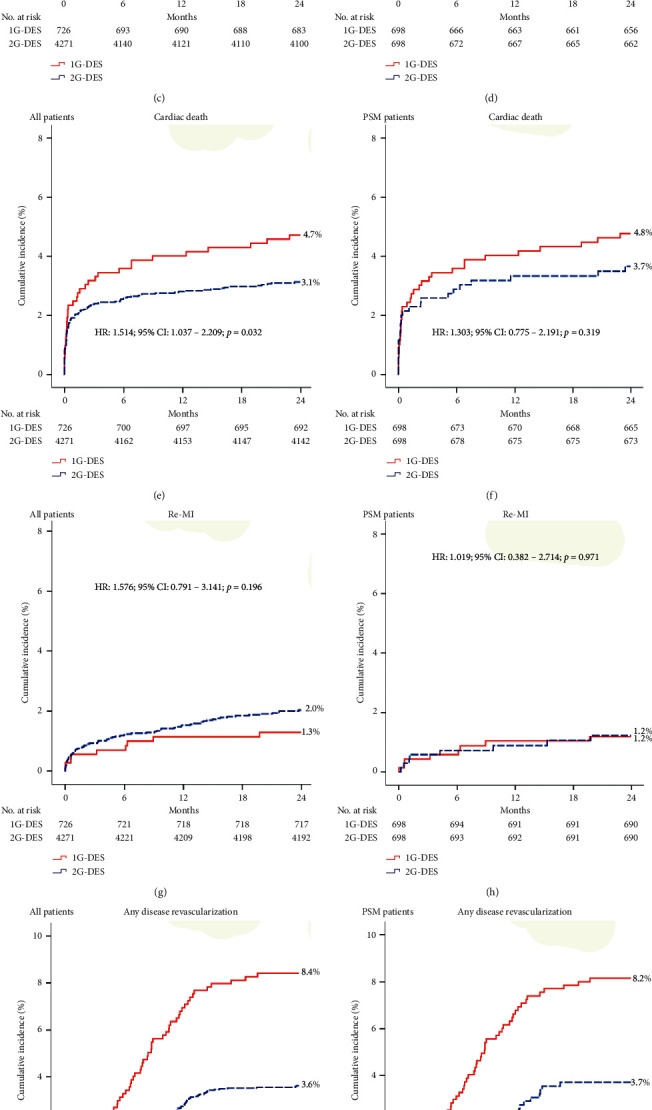
Kaplan–Meier analysis of the incidence rates of POCOs (a, b), all-cause death (c, d), cardiac death (e, f), Re-MI (g, h), any disease revascularization (i, j), and ST (k, l) in all patients (a, c, e, g, i, k) and PSM patients (b, d, f, h, j, l) at 2 years. 1G: first-generation; 2G: second-generation; DES: drug-eluting stent; HR: hazard ratio; CI: confidence interval; POCOs: patient-oriented composite outcomes; Re-MI: recurrent myocardial infarction; ST: stent thrombosis; PSM: propensity score-matching analysis.

The cumulative incidences of major clinical outcomes at 2 years are listed in Table 2 and Figure 2. After PSM analysis, the cumulative incidence rates of POCOs (hazard ratio (HR): 1.467; 95% confidence interval (CI): 1.068–2.015; *p* = 0.018), ADR (HR: 2.259; 95% CI: 1.397–3.654; *p* = 0.001), and ST (HR: 4.361; 95% CI: 1.243–15.30; *p* = 0.021) were significantly higher in the 1G-DES group than in the 2G-DES group. However, the cumulative incidence rates of all-cause death, CD, and Re-MI were similar between the two groups.  Table 3 shows the independent predictors of POCOs and ADR at 2 years. Old age (≥65 years), LVEF of <50%, CPR on admission, use of a lipid-lowering agent, and multivessel disease were significant independent predictors of POCOs. In addition, multivessel disease was a significant independent predictor of ADR in this study.

## 4. Discussion

The main findings of this study are as follows: (1) the cumulative incidence rates of POCOs, ADR, and ST were significantly higher in the 1G-DES group than in the 2G-DES group; (2) the cumulative incidence rates of all-cause death, CD, and Re-MI were similar between the two groups; (3) Multivessel disease was a common independent predictor of both POCOs and ADR.

1G-DES is made of stainless steel with a closed-cell design and has a relatively thick inner diameter (130–150 *μ*m), making them difficult to maneuver through significantly diseased and calcified vessels. In addition, the major serious problem of the 1G-DES was the occurrence of (very) late ST, induced by the polymer or even by the stent material itself [20]. By contrast, the 2G-DES is made of cobalt-chromium (CoCr) and has thinner stent struts (50–90 *μ*m) and showed improved ability for deliverability while maintaining an adequate radial strength [21]. The polymers in the 2G-DES were more biocompatible and thromboresistant than those in the 1G-DES [22] One meta-analysis revealed that CoCr-EESs were associated with significantly lower rates of definite ST than PESs (odds ratio (OR): 0.34, 95% CI: 0.19–0.62) [23]. With regard to diabetes, Bavishi et al. [24] performed a meta-analysis of randomized trials to compare the efficacy and safety between the 1G-DES and the 2G-DES. In their study, the EES showed significantly decreased incidence rates of major adverse cardiac events by 18% (relative risk (RR)): 0.82, 95% CI: 0.70–0.96) and ST by 46% (RR: 0.54, 95% CI: 0.35–0.82) as compared with the 1G-DES. Moreover, the EES showed a trend toward reduced incidence rates of TLR and TVR (*p* = 0.05). The ZES was associated with 89% increased risk of TLR (RR: 1.89, 95% CI: 1.10–3.22) as compared with the 1G-DES in their study. The results of our study may be similar to those of their study. However, their study population was not confined to patients with AMI.

Higher blood glucose level was an important factor of increased risk of death and poor clinical outcome after AMI [25–27]. Kowalczyk et al. [28] reported that patients with HbA1c levels of ≤5.9% had significantly lower posthospital mortality (4.5%) than those with HbA1c levels of >5.9% (25.0%; *p* < 0.001) in 2146 AMI survivors. However, the evident underlying pathological mechanisms related to the adverse clinical outcomes in hyperglycemia status remain unclear. Liu et al. [29] suggested that elevated glucose level is associated with the development of endothelial dysfunction. Moreover, this endothelial dysfunction is the leading cause of platelet activation [30]. In addition, hyperglycemia plays an important role in the development of diabetic macrovascular complications, including atherosclerosis and restenosis [31]. Patients with diabetes have more diffuse disease that often rapidly progresses and tend to have exaggerated neointimal hyperplasia and increased need for repeat revascularization [32]. Moreover, glycosylation of vascular collagen and elastin is thought to lead to a more diffuse pattern of restenosis [33].

Even though the 2G-DES uses an evolved stent platform and a more biocompatible polymer than that in the 1G-DES, data concerning the outcomes between the two DESs in patients with AMI are conflicting [10, 34]. With regard to diabetes, the relative superiority between the 1G-DES and the 2G-DES in patients with diabetes is controversial [35–37]. Furthermore, in patients with prediabetes, limited follow-up data are available regarding the comparative long-term effects of 1G-DES and 2G-DES implantation. Even though the study population, follow-up duration, and definition of prediabetes were different between in our study and that of Kok et al. [38], clinical outcomes were compared between prediabetes and diabetes (11.1% vs. 10.5%). Therefore, the major clinical outcomes of our study could reflect the meta-analysis results of the study of Bavishi et al. [24].

ST is another controversial issue in the DES era. In our study, unexpectedly, the cumulative incidence of very late ST was similar between the two DES generations. By contrast, late ST was significantly different between the two groups. The main cause of these results may be related to the relatively higher numbers of patients who received EES (*n* = 2132/4271, 49.9% ) in this study population. In the study of Bangalore et al. [39], EES was associated with the lowest incidence rates of TVR, Re-MI, and ST as compared with ZES and PES. In the study of Kedhi et al. [35], the difference in the risk of ST was already observed in the early phase and was maintained during the 1-year follow-up. Similarly, in our study, the risk of ST was already determined within 1 year after the index PCI.

Although oral glucose tolerance test (OGTT) is considered more sensitive than measurement of HbA1c level for defining diabetes [15], in this study, prediabetes was determined on the basis of patients' medical history and HbA1c and FPG levels at the index hospitalization. One of the main advantages of measuring HbA1c level is that it can be performed at any time because it does not require fasting. This is especially convenient in circumstances in which performing the test and interpreting the result may be difficult in the milieu of an acute illness such as AMI [38, 40]. Therefore, despite that measurement of HbA1c level may not be ideal, it can be used as an alternative diagnostic tool for making these important assessments.

In this KAMIR study, >50 high-volume university or community hospitals in South Korea participated, but the study population was insufficient to provide meaningful results. Taken together, the results of this study may provide a meaningful message to the interventional cardiologist during PCI to help select the appropriate DES, especially in patients with AMI and prediabetes.

This study has several limitations. First, some data may have been underreported and/or missing owing to the nonrandomized nature of this study. Second, a proportion of the patients with prediabetes (based on HbA1c level) may be diagnosed with overt diabetes if retested using a more sensitive diagnostic test (e.g., OGTT). This may be a source of bias in the study. Third, the study was based on discharge medications, as we could not confirm the participants' adherence or nonadherence to their antidiabetic drugs. Therefore, we could not discern the degree of glycemic control of the participants during the follow-up period, which might constitute an additional bias in this study. Fourth, the 2-year follow-up period was relatively short for determining the long-term major clinical outcomes; thus, longer follow-up period data are required. Fifth, we performed a PSM analysis to strengthen our results, but variables not included in the KAMIR study may have affected the study outcomes.

In conclusion, with regard to the beneficial effects on POCOs, ADR, and ST reduction capacity, in patient with AMI and prediabetes, 2G-DES implantation was more efficacious than 1G-DES implantation during the 2-year follow-up period. However, further randomized trials are needed to more precisely confirm the superior efficacy of 2G-DES over 1G-DES.

## Figures and Tables

**Table 1:** Baseline clinical, laboratory, and procedural characteristics.

Variables	All patients			Propensity score-matched patients		
	1G-DES (*n* = 726)	2G-DES (*n* = 4271)	*p* value	1G-DES (*n* = 698)	2G-DES (*n* = 698)	*p* value
Age (years)	63.8 ± 12.1	64.2 ± 12.4	0.464	63.9 ± 12.0	63.7 ± 12.8	0.814
Men, *n* (%)	535 (73.7)	3169 (74.2)	0.773	516 (73.9)	520 (74.5)	0.854
LVEF (%)	52.6 ± 12.4	52.4 ± 11.2	0.798	52.7 ± 12.54	52.4 ± 11.5	0.618
BMI (kg/m^2^)	24.1 ± 3.1	24.1 ± 3.3	0.854	24.1 ± 2.9	24.1 ± 3.1	0.927
SBP (mmHg)	133.7 ± 24.8	132.3 ± 25.2	0.150	133.2 ± 24.2	134.1 ± 25.5	0.502
DBP (mmHg)	82.0 ± 14.5	80.1 ± 15.0	0.003	81.5 ± 13.9	81.6 ± 15.7	0.866
STEMI, *n* (%)	405 (55.8)	2442 (57.2)	0.484	391 (56.0)	394 (56.4)	0.871
Primary PCI, *n* (%)	376/405 (92.8)	2346/2442 (96.1)	0.003	363/391 (92.8)	371/394 (94.2)	0.452
NSTEMI, *n* (%)	321 (44.2)	1829 (42.8)	0.484	307 (44.0)	304 (43.6)	0.871
PCI within 24 hours	246/321 (76.6)	1563/1829 (85.5)	<0.001	239/307 (77.9)	239/304 (78.6)	0.818
CPR on admission	17 (2.3)	181 (4.2)	0.013	17 (2.4)	16 (2.3)	0.860
Hypertension, *n* (%)	362 (49.9)	2087 (48.9)	0.619	351(50.3)	346 (49.6)	0.789
Dyslipidemia, *n* (%)	70 (9.6)	503 (11.8)	0.095	70 (10.0)	72 (10.3)	0.859
Previous MI, *n* (%)	20 (2.8)	131 (3.1)	0.649	19 (2.7)	15 (2.1)	0.603
Previous PCI, *n* (%)	41 (5.6)	228 (5.3)	0.733	40 (5.7)	33 (4.7)	0.471
Previous CABG, *n* (%)	3 (0.4)	13 (0.3)	0.631	3 (0.4)	2 (0.3)	0.654
Previous CVA, *n* (%)	45 (6.2)	253 (5.9)	0.773	43 (6.2)	37 (5.3)	0.565
Previous HF, *n* (%)	8 (1.1)	48 (1.1)	0.959	8 (1.1)	5 (0.7)	0.579
Current smokers, *n* (%)	293 (40.4)	1875 (43.98)	0.075	280 (40.1)	307 (44.0)	0.143
Peak CK-MB (mg/dL)	127.8 ± 208.2	136.4 ± 196.5	0.278	129.4 ± 210.2	119.5 ± 141.6	0.301
Peak troponin-I (ng/mL)	39.3 ± 88.9	46.1 ± 120.4	0.178	40.4 ± 83.0	39.7 ± 59.3	0.869
NT-proBNP (pg/mL)	2141.2 ± 5100.7	1852.1 ± 4784.8	0.232	2044.5 ± 4062.4	1997.2 ± 3788.8	0.821
hs-CRP (mg/dL)	12.9 ± 36.8	9.7 ± 53.4	0.145	12.1 ± 31.3	14.1 ± 85.9	0.550
Serum creatinine (mg/L)	1.11 ± 1.03	1.10 ± 1.52	0.935	1.11 ± 1.03	1.22 ± 2.77	0.314
Serum glucose (mg/dL)	149.3 ± 48.9	150.4 ± 50.8	0.590	149.1 ± 47.6	151.4 ± 49.9	0.381
Total cholesterol (mg/dL)	188.1 ± 43.4	186.9 ± 44.4	0.494	187.9 ± 43.3	185.8 ± 42.0	0.360
Triglyceride (mg/L)	117.9 ± 75.6	131.9 ± 102.2	<0.001	118.8 ± 75.7	120.3 ± 67.1	0.698
HDL cholesterol (mg/L)	45.0 ± 12.8	43.6 ± 15.4	0.020	44.8 ± 12.3	44.1 ± 15.4	0.380
LDL cholesterol (mg/L)	121.0 ± 36.7	119.5 ± 43.3	0.361	120.7 ± 38.6	118.7 ± 36.1	0.300
Discharge medications						
Aspirin, *n* (%)	684 (94.2)	4119 (96.4)	0.004	658 (94.3)	654 (93.7)	0.653
Clopidogel, *n* (%)	707 (97.4)	3674 (86.0)	<0.001	680 (97.4)	680 (97.4)	1.000
Ticagrelor, *n* (%)	3 (0.4)	363 (8.5)	<0.001	3 (0.4)	4 (0.6)	0.705
Prasugrel, *n* (%)	2 (0.3)	194 (4.5)	<0.001	2 (0.3)	2 (0.3)	1.000
Cilostazol, *n* (%)	217 (29.9)	812 (19.0)	<0.001	197 (28.2)	213 (30.5)	0.378
BB, *n* (%)	573 (78.9)	3552 (83.2)	0.005	552 (79.1)	571 (81.8)	0.200
ACEI, *n* (%)	419 (57.7)	2311 (54.1)	0.071	396 (56.7)	393 (56.3)	0.871
ARB, *n* (%)	178 (24.5)	1101 (25.8)	0.472	174 (24.9)	171 (24.5)	0.901
CCB, *n* (%)	54 (7.4)	240 (5.6)	0.054	48 (6.9)	50 (7.2)	0.917
Lipid-lowering agents, *n* (%)	535 (73.7)	3673 (86.0)	<0.001	530 (75.9)	542 (77.7)	0.447

Infarct-related artery						
Left main, *n* (%)	18 (2.5)	77 (1.8)	0.217	14 (2.0)	168 (2.3)	0.712
LAD, *n* (%)	363 (50.0)	2147 (50.3)	0.878	349 (50.0)	340 (48.7)	0.630
LCx, *n* (%)	134 (18.5)	706 (16.5)	0.199	130 (18.6)	128 (18.3)	0.890
RCA, *n* (%)	211 (29.1)	1341 (31.4)	0.209	204 (29.2)	213 (30.5)	0.590

Treated vessel						
Left main, *n* (%)	25 (3.4)	127 (3.0)	0.495	21 (3.0)	24 (3.4)	0.649
LAD, *n* (%)	443 (61.0)	2537 (59.4)	0.411	424 (60.7)	434 (62.2)	0.582
LCx, *n* (%)	207 (28.5)	1106 (25.9)	0.139	197 (28.2)	191 (27.4)	0.720
RCA, *n* (%)	262 (36.1)	1624 (38.0)	0.320	255 (36.5)	265 (38.0)	0.580

ACC/AHA lesion type						
Type B1, *n* (%)	113 (15.6)	580 (13.6)	0.153	110 (15.8)	115 (16.5)	0.716
Type B2, *n* (%)	237 (32.6)	1367 (32.0)	0.734	226 (32.4)	231 (33.1)	0.776
Type C, *n* (%)	266 (36.6)	1877 (43.9)	<0.001	262 (37.5)	248 (35.5)	0.436

Extent of CAD						
1-vessel, *n* (%)	321 (44.2)	2165 (50.7)	<0.001	303 (43.4)	296 (42.4)	0.705
2-vessel, *n* (%)	224 (30.9)	1326 (31.0)	0.917	217 (31.1)	242 (34.7)	0.154
≥3-vessel, *n* (%)	181 (24.9)	780 (18.3)	<0.001	171 (24.5)	160 (22.9)	0.489

IVUS	177 (24.4)	1007 (23.6)	0.638	172 (24.6)	159 (22.8)	0.413
OCT	0 (0.0)	33 (0.8)	0.017	0 (0.0)	0 (0.0)	—
FFR	1 (0.1)	59 (1.4)	0.004	1 (0.1)	0 (0.0)	0.317

Stents						
SES, *n* (%)	313 (43.1)			303 (43.4)		
PES, *n* (%)	413 (56.9)			395 (56.6)		
ZES, *n* (%)		1466 (34.3)			237 (34.0)	
EES, *n* (%)		2132 (49.9)			366 (52.4)	
BES, *n* (%)		577 (13.5)			73 (10.5)	
Others, *n* (%)		96 (2.2)			22 (3.1)	

Stent diameter (mm)	3.13 ± 0.42	3.13 ± 0.42	0.987	3.13 ± 0.41	3.14 ± 0.43	0.592
Stent length (mm)	25.9 ± 7.15	26.9 ± 11.5	0.031	25.9 ± 7.06	25.9 ± 10.0	0.907
Number of stents	1.54 ± 0.84	1.48 ± 0.80	0.051	1.54 ± 0.80	1.55 ± 0.84	0.740

Values are means ± SD or numbers and percentages. The *p* values for categorical data were obtained from the chi-square or Fisher's exact test. For continuous variables, differences between the two groups were evaluated with independent samples *t*-test. LVEF: left ventricular ejection fraction; BMI: body mass index; SBP: systolic blood pressure; DBP: diastolic blood pressure; STEMI: ST-segment elevation myocardial infarction; NSTEMI: non-ST-segment elevation myocardial infarction; PCI: percutaneous coronary intervention; CPR: cardiopulmonary resuscitation; CABG: coronary artery bypass graft; CVA: cerebrovascular events; HF: heart failure; CK-MB: creatine kinase myocardial band; NT-proBNP: N-terminal pro-brain natriuretic peptide; hs-CRP: high-sensitivity C-reactive protein; HDL: high-density lipoprotein; LDL: low-density lipoprotein; BB: beta-blocker; ACEI: angiotensin-converting enzyme inhibitors; ARB: angiotensin receptor blocker; CCB: calcium channel blockers; LAD: left anterior descending coronary artery; LCx: left circumflex coronary artery; RCA: right coronary artery; ACC/AHA: American College of Cardiology/American Heart Association; CAD: coronary artery disease; IVUS: intravascular ultrasound; OCT: optical coherence tomography; FFR: fractional flow reserve; SES: sirolimus-eluting stent; PES: paclitaxel-eluting stent; ZES: zotarolimus-eluting stent; EES: everolimus-eluting stent; BES: biolimus-eluting stent.

**Table 2:** Clinical outcomes by Kaplan–Meier analysis and Cox proportional hazard ratio analysis at 2 years.

Outcomes	1G-DES	2G-DES	Log-rank	Hazard ratio (95% CI)	*p* value
*All patients*					
POCOs	100 (13.9)	353 (8.8)	<0.001	1.616 (1.294–2.017)	<0.001
All-cause death	43 (5.9)	171 (4.2)	0.035	1.431 (1.024–1.999)	0.036
Cardiac death	34 (4.7)	129 (3.1)	0.030	1.514 (1.037–2.209)	0.032
Re-MI	9 (1.3)	79 (2.0)	0.192	1.576 (0.791–3.141)	0.196
Death or MI	52 (7.2)	244 (6.0)	0.224	1.204 (0.892–1.624)	0.225
^a^Any disease revascularization	58 (8.4)	138 (3.6)	<0.001	2.383 (1.752–3.238)	<0.001
Stent thrombosis (probable or definite)	14 (1.9)	28 (0.7)	0.001	2.956 (1.556–5.615)	0.001
Acute	1 (0.1)	2 (0.0)	0.355	2.941 (0.267–32.44)	0.378
Subacute	4 (0.6)	13 (0.3)	0.290	1.814 (0.591–5.563)	0.298
Late	7 (1.0)	10 (0.2)	0.002	4.146 (1.578–10.89)	0.004
Very late	2 (0.3)	3 (0.1)	0.103	3.967 (0.663–23.74)	0.131

*Propensity score-matched patients*					
POCOs	94 (13.5)	64 (9.5)	0.017	1.467 (1.068–2.015)	0.018
All-cause death	42 (6.0)	36 (5.3)	0.541	1.149 (0.736–1.793)	0.541
Cardiac death	33 (4.8)	25 (3.7)	0.317	1.303 (0.775–2.191)	0.319
Re-MI	8 (1.2)	8 (1.2)	0.971	1.019 (0.382–2.714)	0.971
Death or MI	50 (7.2)	44 (6.5)	0.588	1.118 (0.746–1.677)	0.588
^a^Any disease revascularization	54 (8.2)	24 (3.7)	0.001	2.259 (1.397–3.654)	0.001
Stent thrombosis (probable or definite)	13 (1.9)	3 (0.4)	0.012	4.361 (1.243–15.30)	0.021
Acute	1 (0.1)	0 (0.0)	0.317	—	—
Subacute	3 (0.4)	1 (0.1)	0.316	3.006 (0.313–28.90)	0.340
Late	7 (1.0)	2 (0.3)	0.093	3.526 (0.733–16.98)	0.116
Very late	2 (0.3)	0 (0.0)	0.155	—	—

1G: first-generation; 2G: second-generation; DES: drug-eluting stent; HR: hazard ratio; CI: confidence interval; POCOs: patient-oriented composite outcomes; Re-MI: recurrent myocardial infarction. ^a^Any disease revascularization was composed of target lesion revascularization, target vessel revascularization, and nontarget vessel revascularization.

**Table 3:** Independent predictors for POCOs and any disease revascularization at 2 years in all patients.

Variables	POCOs				Any disease revascularization			
	Univariate		Multivariate		Univariate		Multivariate	
	HR (95% CI)	*p* value	HR (95% CI)	*p* value	HR (95% CI)	*p* value	HR (95% CI)	*p* value
1G-DES vs. 2G-DES	1.616 (1.294–2.017)	<0.001	1.520 (1.213–1.905)	<0.001	2.383 (1.753–3.238)	<0.001	2.228 (1.631–3.043)	<0.001
Age (≥65 years)	1.548 (1.282–1.867)	<0.001	1.318 (1.072–1.620)	0.009	1.024 (0.774–1.355)	0.866	1.109 (0.812–1.515)	0.517
Men	1.269 (1.039–1.550)	0.020	1.030 (0.828–1.282)	0.789	1.097 (0.802–1.501	0.563	1.072 (0.759–1.515)	0.692
LVEF (<50%)	1.652 (1.374–1.986)	<0.001	1.437 (1.191–1.733)	<0.001	1.037 (0.777–1.383)	0.807	1.027 (0.767–1.375)	0.859
Hypertension	1.333 (1.107–1.605)	0.002	1.187 (0.977–1.441)	0.084	1.249 (0.943–1.654)	0.121	1.168 (0.870–1.568)	0.302
Dyslipidemia	1.075 (0.811–1.425)	0.617	1.182 (0.887–1.575)	0.252	1.240 (0.826–1.862)	0.299	1.206 (0.798–1.825)	0.374
CPR on admission	4.004 (3.020–5.309)	<0.001	3.722 (2.799–4.950)	<0.001	1.625 (0.860–3.071)	0.135	1.659 (0.875–3.142)	0.121
Lipid-lowering agent	2.487 (2.038–3.035)	<0.001	2.355 (1.908–2.857)	<0.001	1.503 (1.069–2.114)	0.019	1.514 (1.073–2.137)	0.118
MVD	1.733 (1.430–2.102)	<0.001	1.585 (1.303–1.928)	<0.001	1.852 (1.377–2.491)	<0.001	1.793 (1.328–2.420)	<0.001
ACC/AHA type B2/C lesion	1.141 (0.918–1.419)	0.234	1.131 (0.905–1.413)	0.278	1.014 (0.738–1.392)	0.933	1.062 (0.768–1.469)	0.715
Stent diameter (<3.0 mm)	1.176 (0.967–1.431)	0.104	1.088 (0.891–1.328)	0.407	1.097 (0.812–1.482)	0.548	1.031 (0.760–1.400)	0.843
Stent length (≥28 mm)	1.174 (0.975–1.413)	0.091	1.060 (0.877–1.281)	0.546	1.380 (1.043–1.826)	0.024	1.332 (1.000–1.774)	0.050

POCOs: patient-oriented composite outcomes; HR: hazard ratio; CI, confidence interval; 1G: first-generation; 2G: second-generation; DES: drug-eluting stent; LVEF: left ventricular ejection fraction; CPR: cardiopulmonary resuscitation; MVD: multivessel disease; ACC/AHA: American College of Cardiology/American Heart Association.

## Data Availability

The data used to support the findings of this study are included within the article and supplementary tables.
